# Supplementary material for: Allosteric regulation and crystallographic fragment screening of SARS-CoV-2 NSP15 endoribonuclease
Source: Nucleic Acids Res. 2023 Apr 28;51(10):5255–70. doi: 10.1093/nar/gkad314 (PMC10250223; doi:10.1093/nar/gkad314)
Supplement: gkad314_Supplemental_Files [file gkad314_supplemental_files.zip › Supplementary datasets.zip!/217/Refine_9/01-BUSTER/Cycle-1/shell.01/R_Rfree.html]

# Rxpct factors in resolution bins at cycle 0


|  |  |  |  |  |  |  |  |  |
| --- | --- | --- | --- | --- | --- | --- | --- | --- |
|  | **<d> (Å)**  Rxpct\_Work | Rxpct\_Free | Rxpct\_All | |  |  |  |  |
|  | 16.1098 | 0.3882 | 0.4329 | 0.3897 |  |
|  | 9.3010 | 0.2363 | 0.2823 | 0.2391 |  |
|  | 7.2045 | 0.2090 | 0.2594 | 0.2118 |  |
|  | 6.0889 | 0.2122 | 0.2851 | 0.2154 |  |
|  | 5.3699 | 0.1734 | 0.2397 | 0.1761 |  |
|  | 4.8573 | 0.1621 | 0.1872 | 0.1635 |  |
|  | 4.4681 | 0.1631 | 0.2085 | 0.1654 |  |
|  | 4.1595 | 0.1572 | 0.2304 | 0.1614 |  |
|  | 3.9072 | 0.1713 | 0.2299 | 0.1742 |  |
|  | 3.6958 | 0.1719 | 0.2266 | 0.1744 |  |
|  | 3.5155 | 0.1810 | 0.2208 | 0.1830 |  |
|  | 3.3591 | 0.1859 | 0.2350 | 0.1888 |  |
|  | 3.2220 | 0.1917 | 0.2260 | 0.1936 |  |
|  | 3.1003 | 0.1991 | 0.2189 | 0.2001 |  |
|  | 2.9915 | 0.1982 | 0.2337 | 0.1996 |  |
|  | 2.8934 | 0.2089 | 0.2391 | 0.2105 |  |
|  | 2.8044 | 0.2131 | 0.2495 | 0.2147 |  |
|  | 2.7231 | 0.2145 | 0.2633 | 0.2170 |  |
|  | 2.6484 | 0.2382 | 0.2863 | 0.2413 |  |
|  | 2.5796 | 0.2524 | 0.2767 | 0.2538 |  |
|  | **Overall** | 0.1959 | 0.2405 | 0.1981 |  |

# Rxpct factors in resolution bins at cycle 1


|  |  |  |  |  |  |  |  |  |
| --- | --- | --- | --- | --- | --- | --- | --- | --- |
|  | **<d> (Å)**  Rxpct\_Work | Rxpct\_Free | Rxpct\_All | |  |  |  |  |
|  | 16.1098 | 0.3897 | 0.4342 | 0.3912 |  |
|  | 9.3010 | 0.2358 | 0.2827 | 0.2386 |  |
|  | 7.2045 | 0.2085 | 0.2622 | 0.2115 |  |
|  | 6.0889 | 0.2111 | 0.2831 | 0.2143 |  |
|  | 5.3699 | 0.1723 | 0.2407 | 0.1751 |  |
|  | 4.8573 | 0.1602 | 0.1870 | 0.1618 |  |
|  | 4.4681 | 0.1608 | 0.2064 | 0.1632 |  |
|  | 4.1595 | 0.1551 | 0.2283 | 0.1594 |  |
|  | 3.9072 | 0.1691 | 0.2327 | 0.1722 |  |
|  | 3.6958 | 0.1707 | 0.2304 | 0.1733 |  |
|  | 3.5155 | 0.1795 | 0.2202 | 0.1815 |  |
|  | 3.3591 | 0.1844 | 0.2353 | 0.1874 |  |
|  | 3.2220 | 0.1920 | 0.2251 | 0.1938 |  |
|  | 3.1003 | 0.1985 | 0.2190 | 0.1995 |  |
|  | 2.9915 | 0.1987 | 0.2350 | 0.2001 |  |
|  | 2.8934 | 0.2088 | 0.2407 | 0.2105 |  |
|  | 2.8044 | 0.2131 | 0.2499 | 0.2147 |  |
|  | 2.7231 | 0.2146 | 0.2661 | 0.2172 |  |
|  | 2.6484 | 0.2379 | 0.2879 | 0.2412 |  |
|  | 2.5796 | 0.2521 | 0.2789 | 0.2536 |  |
|  | **Overall** | 0.1948 | 0.2410 | 0.1972 |  |

# Rxpct factors in resolution bins at cycle 2


|  |  |  |  |  |  |  |  |  |
| --- | --- | --- | --- | --- | --- | --- | --- | --- |
|  | **<d> (Å)**  Rxpct\_Work | Rxpct\_Free | Rxpct\_All | |  |  |  |  |
|  | 16.1098 | 0.3937 | 0.4386 | 0.3952 |  |
|  | 9.3010 | 0.2374 | 0.2820 | 0.2400 |  |
|  | 7.2045 | 0.2076 | 0.2623 | 0.2106 |  |
|  | 6.0889 | 0.2093 | 0.2821 | 0.2126 |  |
|  | 5.3699 | 0.1692 | 0.2444 | 0.1723 |  |
|  | 4.8573 | 0.1565 | 0.1881 | 0.1583 |  |
|  | 4.4681 | 0.1574 | 0.2093 | 0.1601 |  |
|  | 4.1595 | 0.1532 | 0.2286 | 0.1575 |  |
|  | 3.9072 | 0.1673 | 0.2353 | 0.1706 |  |
|  | 3.6958 | 0.1694 | 0.2229 | 0.1717 |  |
|  | 3.5155 | 0.1798 | 0.2213 | 0.1818 |  |
|  | 3.3591 | 0.1832 | 0.2365 | 0.1863 |  |
|  | 3.2220 | 0.1915 | 0.2268 | 0.1934 |  |
|  | 3.1003 | 0.1984 | 0.2189 | 0.1994 |  |
|  | 2.9915 | 0.1957 | 0.2332 | 0.1972 |  |
|  | 2.8934 | 0.2081 | 0.2319 | 0.2094 |  |
|  | 2.8044 | 0.2118 | 0.2449 | 0.2133 |  |
|  | 2.7231 | 0.2117 | 0.2697 | 0.2146 |  |
|  | 2.6484 | 0.2307 | 0.2742 | 0.2335 |  |
|  | 2.5796 | 0.2485 | 0.2785 | 0.2502 |  |
|  | **Overall** | 0.1934 | 0.2408 | 0.1958 |  |

# Rxpct factors in resolution bins at cycle 3


|  |  |  |  |  |  |  |  |  |
| --- | --- | --- | --- | --- | --- | --- | --- | --- |
|  | **<d> (Å)**  Rxpct\_Work | Rxpct\_Free | Rxpct\_All | |  |  |  |  |
|  | 16.1098 | 0.3882 | 0.4357 | 0.3898 |  |
|  | 9.3010 | 0.2300 | 0.2824 | 0.2331 |  |
|  | 7.2045 | 0.1977 | 0.2591 | 0.2011 |  |
|  | 6.0889 | 0.1982 | 0.2621 | 0.2010 |  |
|  | 5.3699 | 0.1598 | 0.2492 | 0.1634 |  |
|  | 4.8573 | 0.1503 | 0.1934 | 0.1528 |  |
|  | 4.4681 | 0.1530 | 0.2131 | 0.1560 |  |
|  | 4.1595 | 0.1504 | 0.2248 | 0.1547 |  |
|  | 3.9072 | 0.1644 | 0.2401 | 0.1682 |  |
|  | 3.6958 | 0.1668 | 0.2285 | 0.1695 |  |
|  | 3.5155 | 0.1749 | 0.2130 | 0.1767 |  |
|  | 3.3591 | 0.1802 | 0.2336 | 0.1833 |  |
|  | 3.2220 | 0.1871 | 0.2249 | 0.1892 |  |
|  | 3.1003 | 0.1938 | 0.2089 | 0.1946 |  |
|  | 2.9915 | 0.1919 | 0.2380 | 0.1937 |  |
|  | 2.8934 | 0.2046 | 0.2295 | 0.2059 |  |
|  | 2.8044 | 0.2082 | 0.2327 | 0.2093 |  |
|  | 2.7231 | 0.2105 | 0.2697 | 0.2135 |  |
|  | 2.6484 | 0.2382 | 0.2901 | 0.2415 |  |
|  | 2.5796 | 0.2572 | 0.2900 | 0.2590 |  |
|  | **Overall** | 0.1886 | 0.2399 | 0.1912 |  |
